# First direct measurement of food transit time through the gastrointestinal tracts of plant-eating primates using isotopic tagging

**DOI:** 10.1098/rsos.260875

**Published:** 2026-11-04

**Authors:** Gemma Baker, Lydia K. Greene, James Bendle, Marina B. Blanco, Matthew Allison, Osamu Seki, Sarah E. Greene

**Affiliations:** ^1^ School of Geography, Earth and Environmental Sciences, University of Birmingham College of Life and Environmental Sciences, Birmingham, UK; ^2^ Duke Lemur Center, Durham, NC, USA; ^3^ Department of Biology, Duke University, Durham, NC, USA; ^4^ Institute of Low Temperature Science, Hokkaido Daigaku, Sapporo, Hokkaido Prefecture, Japan

**Keywords:** herbivore ecology, organic geochemistry, lemur, plant biomarkers, *n-*alkanes

## Abstract

The time it takes for food to transit animal gastrointestinal tracts is essential information to fields from organismal evolution to ecosystem ecology. Nevertheless, current estimation methods do not measure food directly, are impractical for many species and often require unnatural conditions to administer. We overcome these limitations by adopting techniques from organic geochemistry. We trace isotopically tagged greens in lemurs, from ingestion to excretion, through isotopic analysis of higher plant biomarkers recovered in faeces. For frugivorous ruffed lemurs (*Varecia rubra*), omnivorous ring-tailed lemurs (*Lemur catta*) and frugo-folivorous sifakas (*Propithecus coquereli*), respectively, the biomarkers first appeared at 1.2, 4.3 and 23.4 h after ingestion and peaked at 2.9, 5.7 and 48.4 h after ingestion. These measurements generally match published estimates from pre-existing proxies. However, not all animals cleared the isotopic signal by the end of sample collection, yielding minimum gut retention times of 23.9–29.5 h for ruffed lemurs, 25.5–30.3 h for ring-tailed lemurs and 95.7 h for sifakas. We thus extend gut retention times approximately threefold from pre-existing proxies. Our interdisciplinary approach is applicable across diverse plant eaters, can help refine models of nutrient, seed and pathogen dispersal and be used to probe consequences of lifestyle and medical interventions on organismal nutrition and health.

## 1. Background

Food transit time, that is the time it takes for food to pass through an animal's gastrointestinal tract—from ingestion to excretion—plays an important role in animal nutrition [1,2,3] and is a critical parameter for modelling faecal dispersal of nutrients, seeds, spores, invasive species, microbes and pathogens [4–7]. Therefore, food transit time mediates the functionality of material exchange between animal and environment in both directions. Multiple approaches have been developed to predict and/or estimate food transit time, including applying general scaling rules (e.g. body mass [6] or intestinal length [8]), tracing undigested particles, such as seeds, bones or spores [5,9], and administering experiments that track artificial proxies for food through the gut, such as liquid dyes, glitter, plastic beads, temperature loggers or element-tagged markers [10–19]. While some methods directly measure particulate excreta of interest, such tracers may be poor proxies for the transit time of materials at the microbial to molecular scale, including digestible foodstuffs. Importantly, different materials and molecules move through the gut at different rates and may be excreted over different spans of time depending on, for example, size and digestive potential; the choice of proxy should be suitable for the question(s) of interest. Many of these proxies cannot be validated or used in certain species, require unnatural conditions to administer (e.g. anaesthesia [10]) or involve the introduction of substances that may not be fully recovered [15,20], particularly in wild populations [16,17] – all of which may confound proxy functionality.

Here, we overcome these challenges by piloting a new method that adopts established techniques from organic geochemistry to directly track the time window over which plant food is ingested and excreted ( figure 1). Differences in stable isotopic ratios across biological and geological substrates have long been exploited by multiple fields of natural science to track the movement of materials and elements through space and time. Increasingly, geochemists deploy ‘compound-specific’ stable isotope measurements, that is the isotopic ratio of biomarkers (particular organic compounds with known origins) to track biogeochemical cycling with greater specificity. We propose the use of *n*-alkanes, that is leaf waxes, as an ideal biomarker target for measuring the transit time of plant material from animal ingestion to excretion. High molecular weight *n*-alkanes are produced almost exclusively by higher plants, are strongly resistant to degradation, survive in geological sediments for millions of years [21] and passively transit through animal guts [22,23], upon which their isotopic composition can be measured.

**Figure 1.**
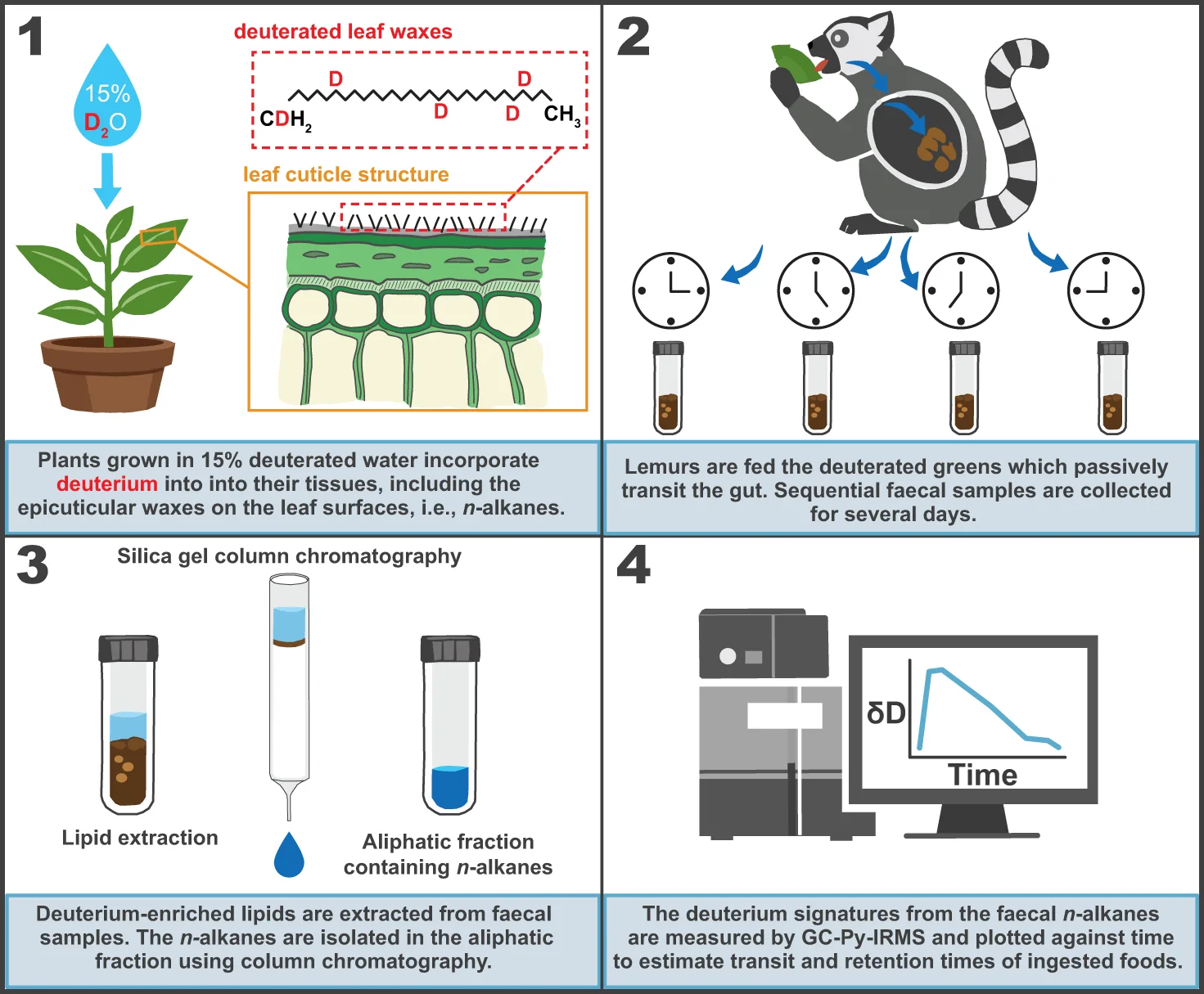
An overview of the methods employed for this study.

We pilot this method by growing collard greens in 15% deuterated water, creating plant tissues, including *n-*alkanes, ‘tagged’ with artificially extreme hydrogen isotopic signatures (*δ*D). We fed the deuterated greens to captive lemurs and collected baseline and sequential faecal samples for 2–5 days. Our focal species are ruffed lemurs (*Varecia rubra*), ring-tailed lemurs (*Lemur catta*) and Coquerel's sifakas (*Propithecus coquereli*) (figure 2). Ruffed lemurs are highly frugivorous [24,25] and have a short, simple gastrointestinal tract [26]. Estimates of food transit times from seed-passage studies in Madagascar [27,28], acetate beads and element-labelled markers in captivity [10,14,29] show a range from 42 min to 8.5 h. Sifakas, in contrast, are more folivorous [30,31] and rely on a long, complex gastrointestinal tract [26] and specialized gut microbiome [32] for digestion. Estimates of food transit times, from element-labelled markers in captivity [10,20,29], show a range from 17 to over 48 h, with not all animals clearing larger proxies at the end of data collection [10]. For ruffed lemurs and sifakas, researchers have sought to quantify both the first and last appearance of the markers, that is gut transit and gut retention times; however, maximum durations have been particularly hard to pinpoint in the folivores. Omnivorous ring-tailed lemurs are an intermediary between these two extremes [26]. Estimates of food transit times from plastic markers in captivity range from 1.5 to 5.6 h [33].

**Figure 2.**
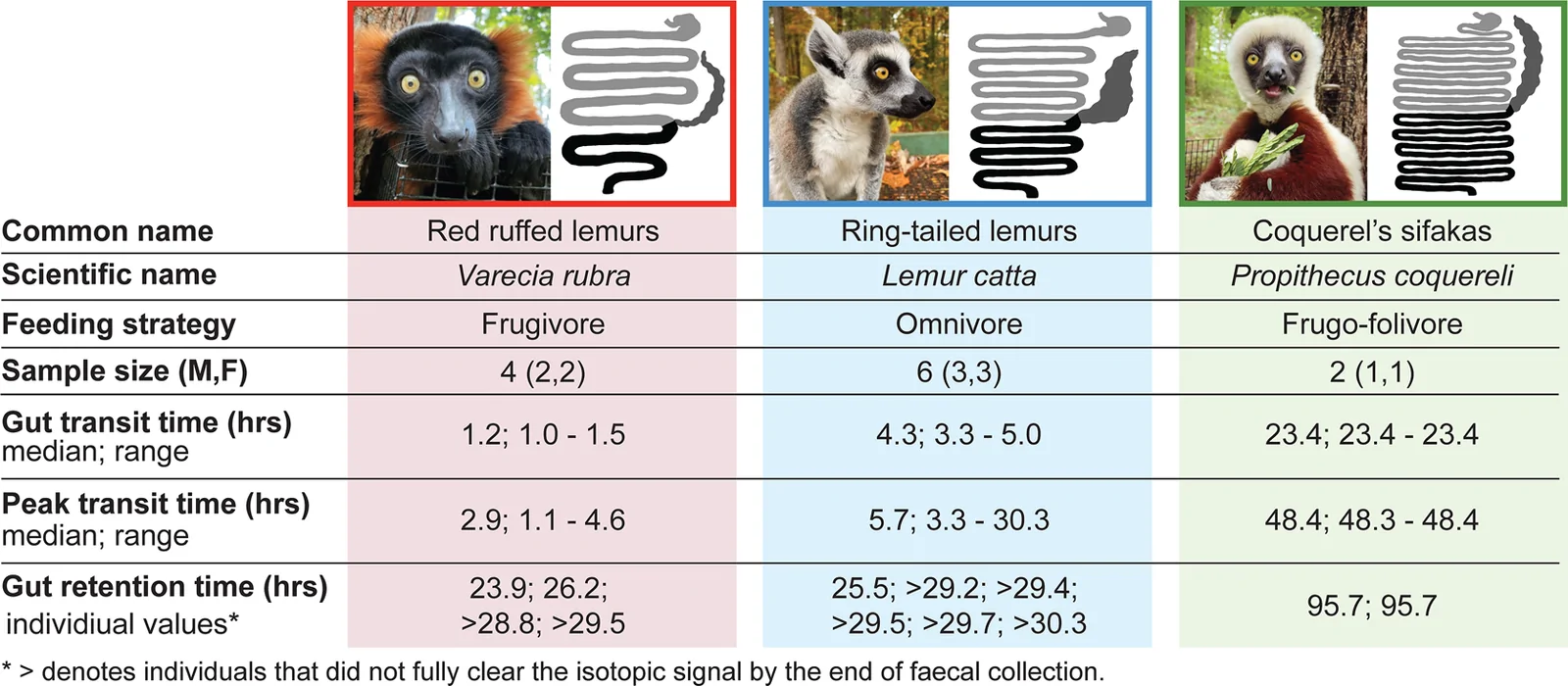
Overview of study species, including gastrointestinal tract illustrations, sample sizes, results of gut and peak transit times per species and retention times for each individual. Photos by L.K.G.; illustrations by Sally Bornbusch.

We expect that once the deuterated collard greens are consumed and transit the lemurs’ guts, there will be a clear and temporary increase in faecal *n*-alkane *δ*D, as measured using gas chromatography-isotope ratio mass spectrometry (GC-IRMS). We expect our method to capture the full transit-time window for each species. We define gut transit time as the first appearance of an artificial isotopic signal in the faecal *n*-alkanes, peak transit time as the maximum abundance of the artificial isotopic signal in the faecal *n*-alkanes and gut retention time as the duration after which faecal *n*-alkanes return to baseline isotopic composition. Based on the species’ different gut morphologies, feeding ecologies and published transit-time estimates, we predict gut transit, peak transit and gut retention times to all be shortest in ruffed lemurs, intermediate in ring-tailed lemurs and longest in sifakas.

## 2. Methods

The methodology for this study is summarized in figure 1.

### 2.1. Isotopic labelling of plant food material

Collard greens (*Brassica oleracea*)—a staple in the diets provisioned to the study lemurs—were sown and germinated by expert staff at the Research Greenhouse in the Department of Biology at Duke University within two weeks during the autumn of 2023, with greater than 80% germination success. After germination, 36 seedlings were transplanted into 7.57 l (2 gallon) pots. After one week, 15–15–15 fertilizer treatment was applied three times weekly to promote leaf growth. No pesticide treatment was applied.

Approximately six weeks after sowing, the heavy water (D_2_O) treatment was started. Specifically, 1.5 l of heavy water was diluted with 8.5 l of regular (tap) water to produce 10 l of 15% concentration D_2_O. A total of 333 ml per plant, in 111 ml doses, was applied to the established plants over five weeks to force the plants to incorporate the deuterium into the plant tissues. This deuterium concentration was chosen to be sufficient to label plant material, but not high enough to significantly inhibit plant growth and development [34]. To limit the loss of deuterium oxide by evaporation, transpiration and run-off, and increase uptake by the plants, they were grown in small pots on trays for run-off capture. Plants were harvested 1–7 days prior to the feeding event and stored in a refrigerator at 5°C between harvest and the feeding experiment. Two samples of the isotopically labelled collard greens were taken for isotopic analysis to confirm that the plant tissues were sufficiently deuterated (see below).

### 2.2. Subject animals and housing

The subject animals included 12 healthy adult captive lemurs (aged 2–22 years) in six mixed-sex social pairs, including two pairs of ruffed lemurs, three pairs of ring-tailed lemurs and one pair of sifakas, housed at the Duke Lemur Center (DLC) in Durham, North Carolina. The animals were all individually recognizable by unique markings, coloured collars or tail shaves. At the time of study, the animals were housed in large indoor stalls and given access to outdoor yards when temperatures were reliably above 5°C.

The subjects received a once-daily provisioned diet in the morning. The ruffed and ring-tailed lemurs were fed a standard primate biscuit (Lab Diet no. 5037 Monkey Diet) and a rotating mixture of fresh fruit and vegetables daily. The sifakas received folivore chow (Mazuri Leaf-Eater Primate Diet Mini-Biscuit, no. 5672), nuts or beans, sweet potato or corn, a mixture of fresh vegetables, and kale or collard greens daily. This fare was supplemented in the afternoon with local foliage, harvested from nearby forests and rinsed in 10% bleach. Water was freely available at all times.

### 2.3. Study design and sampling

We administered the labelled greens to each lemur once between November and December of 2023, focusing on one social pair at a time. To do so, we created a smoothie comprising 50 g of the labelled collard greens mixed with preferred food items (fresh banana for the ruffed lemurs, banana and strawberry jam for the ring-tailed lemurs and peanut butter for the sifakas). In addition, we mixed 1 g of Wilton Icing Colour Paste in royal blue into the smoothie for the males, in an attempt to differentiate faecal samples excreted overnight when observations were not possible.

Prior to feeding out normal morning diets, DLC research staff separated the pair into adjacent enclosures and presented the smoothie in pre-weighed bowls. If required, further food incentives were used to encourage inspection of the smoothie, involving banana-flavoured baby food (for the ring-tailed lemurs) or peanut butter (for the sifakas). The ruffed lemurs required no incentives. After 30 min, the bowls were removed and re-weighed to calculate the volume consumed, and the pair was reunited. The ring-tailed and ruffed lemurs consumed between 31 and 100% of the greens provided, with the two sifakas consuming only approximately 20% each. Nevertheless, these volumes were sufficient to produce a measurable spike in faecal isotopic signal for all animals.

We collected one faecal sample per animal within 7 days prior to the feeding of the smoothie to establish the ‘baseline’ deuterium signature (*δ*D). We then collected serial faecal samples in the hours and days post smoothie ingestion, noting individual identity, date and time. If the food dye was visible in the faeces, we used it to identify samples in the early morning produced during non-observation hours and took the midpoint of the non-observation (overnight) period as the estimated time of defecation. For the ruffed and ring-tailed lemurs, we collected sequential samples for 2–3 days, which covers a period far longer than published estimates of transit time in either species [10,14,27–29,33]. Bolstering this, after 2–3 days, the food dye was no longer visible. For the sifakas, the food dye was never visible in faecal samples. Therefore, we collected one faecal sample per day per animal for the first two focal days, and at every observed faecal event after for five consecutive days. We did not collect any overnight samples for this species.

Using this regimen, we collected 206 samples with an average of 17 per individual. Samples were scooped from the enclosure floor into sterile Whirl-Pak sample bags and put immediately on ice. They were frozen at −80°C within 2 h of defecation.

### 2.4. Sample processing

We dehydrated plant and faecal samples on site at the DLC using commercial food dehydrators, including a final stint at greater than 56°C to comply with UK import licence. We then wrapped samples in clean aluminium foil and placed them into individual sterile bags. We shipped samples to the University of Birmingham, UK, under IV58 licence (license number ITIMP22.1129) and stored them in a freezer at −20°C until analysis.

We selected for analysis a subset of 103 faecal samples (average: eight samples per animal; range: 7–9 per animal) that spanned the entire collection window for each animal. The baseline sample, first appearance of blue dye and last appearance of dye (if applicable) were all analysed. The remaining samples selected were chosen systematically between these. For no animal was the final faecal sample with elevated deuterium produced overnight. We processed samples according to established protocols [23] using solvents of high-performance liquid chromatography grade. In brief, we dehydrated and ground plant and faecal samples into a fine powder before extracting approximately 1 g material by ultrasonicating three times with 10 ml dichloromethane : methanol in a 9 : 1 (v/v) ratio to generate a total lipid extract (TLE). We then used column chromatography using silica gel as the solid phase to separate the TLE into four fractions, of which the first (eluted with 100% *n*-hexane) contained aliphatic hydrocarbons, that is the *n*-alkane targets of this study.

### 2.5. Sample analysis

We subsequently analysed extracted *n*-alkanes using an Agilent 7890B GC-flame ionization detection machine to determine their concentrations with an external *n*-alkane standard of known concentration. The front inlet and back detector were held at 330°C throughout. Compressed air was the carrier gas. An Agilent DB1 column (60 m length, 0.25 mm internal diameter, 0.25 μm film thickness) with a 100% dimethylpolysiloxane stationary phase was used. The oven temperature programme held at 70°C for 1 min, before ramping to 130°C at 30°C min^−1^, then to 320°C at 4°C min^−1^, holding for 10 min, for a total run time of 60.5 min.

The deuterium isotope composition of the individual *n*-alkanes was measured using a GC coupled to a pyrolysis furnace and IRMS (GC-Py-IRMS). The system consisted of an Agilent 7890B GC connected via a high-temperature conversion interface (Py) operated at 1450°C to an Elementar isoprime visION IRMS. Separation was achieved using a DB-5 fused silica capillary column (60 m length, 0.25 mm internal diameter, 0.25 μm film thickness). The GC oven temperature was initially held at 50°C, ramped at 20°C min^−1^ to 120°C, then at 5°C min^−1^ to 310°C, and held isothermally for 25 min. Hydrogen isotope values were calibrated using the Indiana University *n*-alkane mixture B4 as the reference material. All *δ*D values were reported relative to the Vienna Standard Mean Ocean Water (VSMOW) standard. We analysed each sample in duplicate to ensure reproducibility and calculated a standard deviation (measurement error). Collection and analysis of baseline samples allowed for the calculation of C_29_*δ*D values relative to the baseline for each conspecific where the baseline C_29_*δ*D value was subtracted from the sample C_29_*δ*D value. Relative C_29_*δ*D values allowed individuals to be directly compared with one another.

### 2.6. Data analysis

For all animals, we calculated the time of first appearance (hereafter ‘gut transit time’), maximal appearance (hereafter ‘peak transit time’) and the time of last appearance (hereafter ‘gut retention time’). For the deuterium *n*-alkane isotopes, we determined gut transit time to be when the *δ*D signature increased by more than 5% from baseline or ‘normal’ conditions. We define peak transit time as the time when the *δ*D signature was at its highest (peak). We define gut retention time as the time of last appearance of the elevated *δ*D marker, i.e. when the *δ*D signature returned to values within, or less than, 5% of the *δ*D signature of the baseline sample for that individual. We present summary statistics for gut transit, peak transit and gut retention times within species.

## 3. Results

### 3.1. Deuterated collard greens

We successfully grew collard greens with an artificial, extreme hydrogen isotopic signature (*δ*D) using 15% D_2_O. Indeed, the two samples of collard greens harvested prior to animal experimentation had C_29_ *n*-alkane *δ*D values of 5817 ± 7.4 and 7934 ± 194.5‰.

### 3.2. Estimates of food transit time

All study lemurs consumed sufficient isotopically enriched plant matter, with faecal *n*-alkanes demonstrating clear isotopic enrichment in the hours to days following the ingestion of isotopically tagged plant material (figure 3). The maximum observed isotopic enrichment of the representative C_29_ *n*-alkane (the most consistently measurable *n*-alkane homologue) varied between 500 and 7000‰ VSMOW relative to baseline composition. The maximum faecal *n*-alkane isotopic enrichment for each lemur species directly reflected the mass of labelled collard greens consumed (supplementary material, table S1).

**Figure 3.**
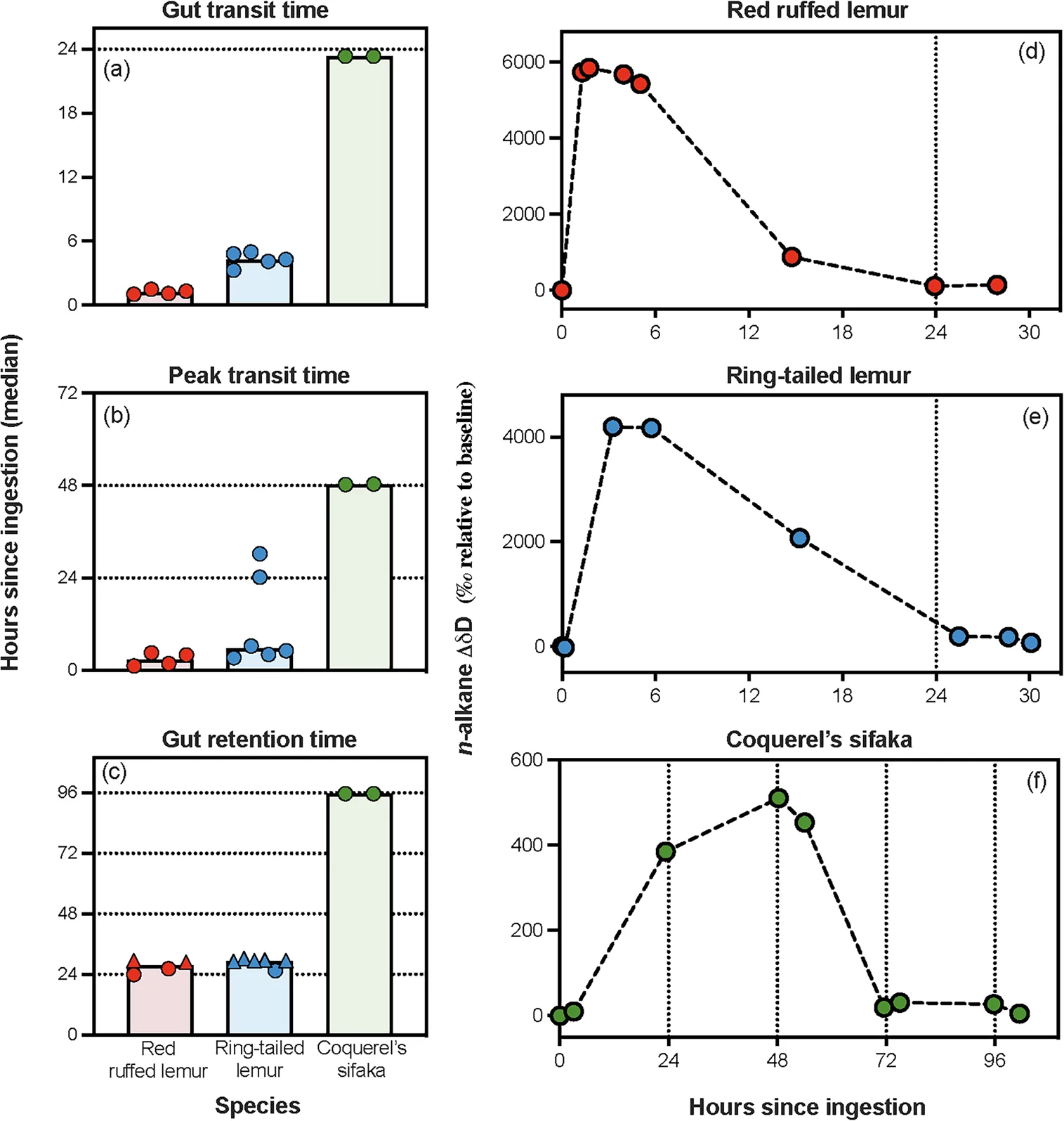
Median (a) gut transit time, (b) peak transit time and (c) gut retention time for red ruffed lemurs (red), ring-tailed lemurs (blue) and Coquerel's sifakas (green). Circles and triangles in (c) depict animals that, respectively, did and did not fully clear the isotopic signal by the end of faecal collection. Note (a) excludes the single value of 15.3 h that was probably overestimated. Schematics of transit times for exemplar individuals: (d) red-ruffed lemur, (e) ring-tailed lemur and (f) Coquerel's sifaka where the *y*-axis represents the *δ*D values of the C_29_ *n*-alkane homologue relative to baseline. Dotted lines demarcate days.

For the ruffed lemurs, median gut transit time was 1.2 h with a range of 1.0–1.5 h (figure 3a). For five of the six ring-tailed lemurs, median gut transit time was 4.3 h with a range of 3.3–5.0 h. The sixth ring-tailed lemur produced no faecal samples from 1 h post ingestion until the end of the observation session. Our estimate of her gut transit time stems from an overnight sample and is 15.3 h (i.e. the midpoint of the non-observation (overnight) period), which is very likely an overestimate and should be interpreted with caution. For both sifakas, gut transit time was 23.4 h.

For the ruffed lemurs, median peak transit time was 2.9 h with a range of 1.1–4.6 h (figure 3b). For the ring-tailed lemurs, median peak transit time was 5.7 h with a range of 3.3–30.3 h. For the two sifakas, peak transit times occurred at 48.3 and 48.4 h.

Surprisingly, only two ruffed lemurs and one ring-tailed lemur fully cleared the isotopic signal by the end of sample collection. After 2–3 days, the other conspecifics were still excreting *δ*D values elevated from baseline (figure 3c). We thus provide gut retention times for these two ruffed lemurs at 23.9 and 26.2 h (figure 3d) and for the ring-tailed lemur at 25.5 h (figure 3e). For the remaining animals, we used the time of last sampling to estimate minimum gut retention times of 28.8–29.5 h for the ruffed lemurs and 29.2–30.3 h for the ring-tailed lemurs. Both sifakas fully cleared the isotopic signal at the same time, yielding a gut retention time of 95.7 h or 4 days (figure 3f).

## 4. Discussion

By feeding isotopically tagged greens to lemurs and tracking isotopic signals in subsequent faecal samples, we provide a novel approach to directly measure food transit time through animal guts. Unlike artificial proxies of food, the *n-*alkanes measured herein for their  *δ*D values derived directly from the plant foods themselves, which incorporated the heavy hydrogen from D_2_O into their tissues. Unlike bulk isotopes, which could reflect material not only from the diet but also from intestinal epithelia and gut microbiota, the compounds we targeted can only derive from higher plants. As expected, we find that species with simpler versus more complex gastrointestinal tracts, respectively, exhibit faster and slower gut transit and peak transit times, measurements that are broadly consistent with previous estimates from these species; by contrast, we demonstrate that pre-existing methods underestimate gut retention time by a factor of three for both ruffed lemurs and sifakas [10,14,20,27–29]. We publish, to the best of our knowledge, the first measurements of gut retention time in ring-tailed lemurs.

It is not a surprise that directly measuring food transit captures longer gut retention times, outperforming proxies such as liquid dyes and foreign solid objects. Compared to liquid dyes, which pass through the gut more quickly than do solids [35] and become diffuse and harder to detect with passing time (particularly for animals with more complex gastrointestinal tracts like sifakas), isotopic tagging robustly pinpoints the ‘tail’ end of food transit times. Compared to foreign objects [12], labelled plant material adds bulk to the gut and offers nutritional value to both the animal and its symbiotic gut microbes, the latter fermenting fibres, which, in turn, may mediate retention times [2,36]. The success of this method, which measures food directly without disrupting natural behaviours or introducing foreign items, has applications to individual healthcare, the ecology of diverse plant eaters consuming a range of edible plants and models of faecal dispersal.

At the ecological and landscape scales, faeces are a major mechanism by which materials and microorganisms cycle through ecosystems, particularly for animals that are mobile, have large biomass, aggregate in large groups and/or migrate long distances [7,37–39]. The gut transit, peak transit and gut retention times are essential parameters to quantitatively model the dispersal or cycling of various nutrients, elements, seeds, spores, invasive species and microbes, including pathogens [4,6]. For modellers interested in questions of dispersal, it is critical to consider whether gut transit, peak transit or gut retention time (or another metric) is the most meaningful parameter for a given ingested material, study question and study system. For studies targeting nutrient or seed dispersal predictions, peak transit times, or the window of time over which the maximal signal is excreted, may be the most valuable measurement upon which to build a dispersal model. By contrast, for studies seeking to quantify the maximum dispersal potential of (e.g.) invasive species or pathogens that can be biologically relevant even in trace quantities, retention times, which are more difficult to estimate using pre-existing methods, may be required. Indeed, we anticipate that gut transit time—the metric most robustly captured by pre-existing methods—is likely to be the parameter of least relevance for dispersal predictions. With enough sample density, our method could be extended to provide probability density functions; such distributions would be the ideal input for a universally applicable statistical model of dispersal for any faecally dispersed material.

Beyond ecology, accurate transit time estimates can improve understanding of how lifestyle and medical interventions affect axes of health. In humans, an established literature highlights relationships between transit time and variables such as diet [13], exercise [40], the gut microbiome [2,41], glucose homeostasis [1] and certain cancers [42]. Similar relationships probably exist for other animal species. More reliable transit times could help tune targeted therapies for humans and wildlife, especially for dietary optimization and nutrient absorption, and advance gut microbial therapies like faecal transplants that alleviate gastrointestinal distress or dysbiosis in sick individuals [43]. Such therapies may depend on knowing the normal range of variation between conspecifics. Notably, ring-tailed lemurs (the species for which we had the largest sample size) showed the greatest food transit time variation among individuals, even within social pairs with synchronized daily rhythms, heterogeneity that could stem from their omnivorous diets and/or intraspecific variation in metabolism or gut morphology. It is clear that longer experiments are needed to fully capture gut retention times, and more robust metadata (e.g. behavioural monitoring and individual gut length) are required to unpick the causes of conspecific variability. Accurate food transit times may be of further value to the design of *in vitro* studies on food digestibility and microbial action [44]. In summary, our method has numerous potential applications beyond ecology by improving transit and retention time accuracy while avoiding unnatural experimental conditions that may introduce confounding variables.

While we overcome drawbacks of pre-existing transit-time proxies, there are important limits to the applicability of our method. First, our method is intended for plant eaters only and may be workable only for animals of intermediate size. Too small, like insects, and there may be insufficient faecal mass for analysis. Too large, like elephants, and it may become costly to grow sufficient plant material to induce a measurable isotopic signal in faecal *n*-alkanes. The total achievable faecal deuterium spike depends not only on the mass of crop grown but also on the achieved deuterium concentration of its *n*-alkanes, the mass of ingested crop and the dilution of the deuterated *n*-alkanes by other ingested leafy items. Differences in individual or species feeding behaviours, as well as ethics approval to withhold other dietary items, could further influence study design and proxy sensitivity.

We have only attempted this method so far with one type of domesticated crop (collard greens) grown in water at a single deuterium concentration. Some plants or plant tissues may not tolerate deuterated water or may grow too slowly to be practical. While this method is clearly promising, further work is needed to ascertain the limits of its potential applicability across species and conditions. Whereas captive animals at accredited institutions are perhaps best suited for this method, it is important to consider that captive animals often eat different diets and have different activity levels relative to their wild kin, both of which could mediate food transit times. Our method may be challenging to administer to wild populations, unless they are habituated to close observation and provisioned feeding. Lastly, our method adds analytical complexity and costs compared with faecal proxies that can be visually identified, like dye and glitter. Nonetheless, the methodological advantages, where applicable, are clear.

## 5. Conclusions

Bulk isotope ratios have a rich history of applications to the fields of geology, evolution and ecology. By contrast, ecologists have been slower to adopt established geochemical approaches using compound-specific stable isotopes [45], perhaps owing to lack of awareness of their power and potential. We show here just one application, using the compound-specific isotopic ratio of labelled leaf waxes to track gastrointestinal passage of plant food, allowing us to isolate and chemically fingerprint a particular class of dietary items from a faecal sample. Closer interdisciplinary working between ecologists and geologists can capitalize on the potential of geochemical techniques to address critical questions in organismal evolution and ecology.

## Supplementary Material



## Data Availability

Data for this manuscript is archived at https://doi.org/10.25500/edata.bham.00001343. Supplementary material is available online [46].
