# Supplementary material for: First direct measurement of food transit time through the gastrointestinal tracts of plant-eating primates using isotopic tagging
Source: R Soc Open Sci. 2026 Nov 4;13(11):260875. doi: 10.1098/rsos.260875 (PMC13730660; doi:10.1098/rsos.260875)
Supplement: Supplementary file 1 [file rsos.260875.zip › rsos.260875/rsos260875_si_001.docx]

**First direct measurement of food transit time through the gastrointestinal tracts of plant-eating primates using isotopic tagging**

Gemma Baker^a,b^*^†^, Lydia K. Greene^c,d†^, James Bendle^a,b^, Marina B. Blanco^c,d^, Matthew Allison^a^, Osamu Seki^e^, Sarah E. Greene^a,b^

^a^School of Geography, Earth and Environmental Sciences, University of Birmingham

^b^Birmingham Institute of Forest Research, University of Birmingham

^c^Department of Biology, Duke University

^d^MadaGap, Inc.

^e^Institute of Low Temperature Science, Hokkaido University

*Corresponding author: gxb760@student.bham.ac.uk

^†^These authors contributed equally

**SI Supplementary Information**

**S1 - Methods**

Deuterated greens were incorporated into ‘smoothies’ comprising 50 g of the labelled food material and different food items based on the preferences of the individual species (peanut butter for the sifakas, banana for the ruffed lemurs, and banana and strawberry jam for the ring-tailed lemurs). Smoothies were weighed before and after they were presented to the lemurs so that the volume of labelled food consumed could be calculated (Table S.1).

Table S.1. Volume of deuterium-labelled collard greens (*Brassica oleracea*) consumed by the subject animals of this study.

| Species | Lemur | Labelled collard greens consumed | |
| --- | --- | --- | --- |
|  |  | g | % |
| Coquerel’s sifaka (*Propithecus coquereli*) | Thrax | <10 | <20 |
|  | Furia | <10 | <20 |
| Red ruffed lemur (*Vareca rubra*) | Afo | 50.00 | 100.00 |
|  | Judith | 50.00 | 100.00 |
|  | Hunter | 49.54 | 99.08 |
|  | Pandora | 49.62 | 99.24 |
| Ring-tailed lemur (*Lemur catta*) | Schweppes | 15.50 | 31.01 |
|  | Sprite | 39.17 | 78.33 |
|  | Jones | 25.45 | 50.90 |
|  | Thea | 19.30 | 38.59 |
|  | Onyx | 26.51 | 53.02 |
|  | Hibernia | 13.91 | 27.81 |

**Sample Analysis**

Samples were analysed in duplicate for the C29 𝛿D values and a standard deviation representing machine/measurement error was calculated in each case. The average of the two 𝛿D values is presented. Where the one of the analyses failed, no error is presented and no average could be calculated.

To enable direct comparison of C29 𝛿D values between individuals, a ‘relative’ 𝛿D value was calculated from the baseline sample obtained prior to administering the smoothie using the below equation.

$$Relative C_{29} \delta D= Sample C_{29} \delta D-Baseline C_{29} \delta D$$
